# Distinct decision-making properties underlying the species specificity of group formation of flies

**DOI:** 10.1098/rsos.220042

**Published:** 2022-08-24

**Authors:** Riku Shirasaki, Ryoya Tanaka, Hiroki Takekata, Takashi Shimada, Yuki Ishikawa, Azusa Kamikouchi

**Affiliations:** ^1^ Graduate School of Science, Nagoya University, Nagoya, Aichi 464-8602, Japan; ^2^ Center for Strategic Research Project, University of the Ryukyus, 1 Senbaru, Nishihara, Okinawa 903-0213, Japan; ^3^ Mathematics and Informatics Center, The University of Tokyo, Tokyo, Japan; ^4^ Department of Systems Innovation, Graduate School of Engineering, The University of Tokyo, Tokyo 113-8656, Japan; ^5^ Graduate School of Life Sciences, Tohoku University, Miyagi 980-8577, Japan

**Keywords:** *Colocasiomyia alocasiae*, group size, group formation process, grouping behaviour, group size dependency

## Abstract

Many animal species form groups. Group characteristics differ between species, suggesting that the decision-making of individuals for grouping varies across species. However, the actual decision-making properties that lead to interspecific differences in group characteristics remain unclear. Here, we compared the group formation processes of two Drosophilinae fly species, *Colocasiomyia alocasiae* and *Drosophila melanogaster*, which form dense and sparse groups, respectively. A high-throughput tracking system revealed that *C. alocasiae* flies formed groups faster than *D. melanogaster* flies, and the probability of *C. alocasiae* remaining in groups was far higher than that of *D. melanogaster*. *C. alocasiae* flies joined groups even when the group size was small, whereas *D. melanogaster* flies joined groups only when the group size was sufficiently large. *C. alocasiae* flies attenuated their walking speed when the inter-individual distance between flies became small, whereas such behavioural properties were not clearly observed in *D. melanogaster*. Furthermore, depriving *C. alocasiae* flies of visual input affected grouping behaviours, resulting in a severe reduction in group formation. These findings show that *C. alocasiae* decision-making regarding grouping, which greatly depends on vision, is significantly different from *D. melanogaster*, leading to species-specific group formation properties.

## 1. Introduction

Group formation is an important survival strategy in many animals. Animals benefit from grouping in various ways, including increased avoidance of predation, more efficient foraging, mate finding and reduced energy costs for migration (reviewed in [1]). There are also costs associated with grouping such as resource competition and disease transmission. Differences in the balance of the costs and benefits of grouping lead to species-specific group characteristics.

Decision-making at the individual level (joining, staying or leaving the group) is a key factor influencing group formation. Species-specific group characteristics thus partly rely on species-specific decision-making properties that determine their behaviours. However, how individual decision-making properties differ across species with distinct group characteristics remains unclear. To address this question, a comparison of the group formation processes in related species with different group characteristics is required.

**Figure 1.**
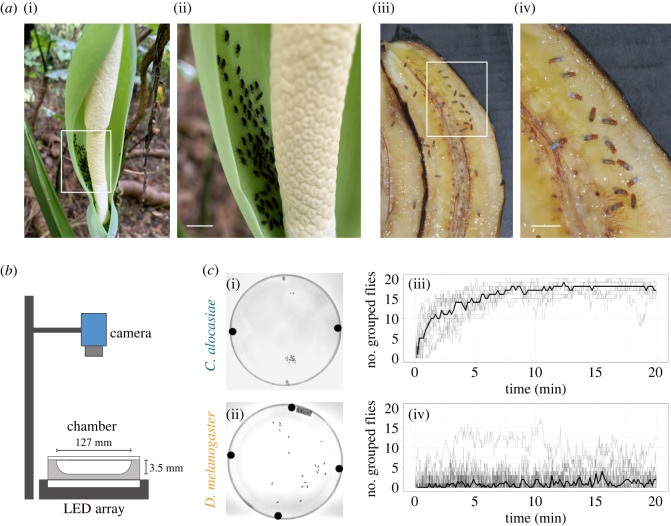
Grouping behaviours of *Colocasiomyia alocasiae* and *Drosophila melanogaster* under natural and artificial conditions. (*a*) *C. alocasiae* flies form a dense group on inflorescences of their host plant *A. odora* (i,ii). *D. melanogaster* flies also form groups rather than disperse randomly on host fruit (iii,iv). Scale bars, 5 mm. (*b*) Experimental set-up under laboratory conditions. (*c*) Group formations of two fly species. Data on *C. alocasiae* (i,iii) and *D. melanogaster* (ii,iv) are shown. Examples of fly distributions at 20 min following experiment start (i,ii). Time traces of the numbers of grouped flies (iii,iv). Thick lines indicate the median of the time traces while thin lines show the individual traces for each chamber.

The fly species *Colocasiomyia alocasiae* (Okada) (Diptera: Drosophilidae) is found in dense groups on the inflorescences of their host plant, *Alocasia odora* K. Koch (Araceae) (figure 1*a*) [2]. *A. odora* provides breeding sites and adult food for this fly species, and its oviposition and larval growth depend on inflorescences [2–4]. *Drosophila melanogaster*, a related fly species belonging to the same subfamily Drosophilinae as *C. alocasiae*, has been widely used as a model animal in neuroethology as well as in other research fields. *D. melanogaster* flies are known to form groups rather than disperse randomly on both host fruit and artificial chambers without fruit (figure 1*a*) [5–11]. Recently, the group formation process was explored in detail in *D. melanogaster* [12], facilitating comparisons with equivalent processes in *C. alocasiae* flies.

In this study, we established a behavioural assay to compare the group formation processes of *C. alocasiae* and *D. melanogaster*. We demonstrated distinct decision-making properties of individuals regarding group formation between these two fly species, which reflects differences in species-specific group characteristics.

## 2. Material and methods

### 2.1. Experimental animals

The inflorescences of *A. odora*, which house *C. alocasiae* eggs and larvae, were collected from the Nishihara Campus of the University of the Ryukyus (26°14′51.3″ N 127°45′54.5″ E) on Okinawa Main Island, Japan, from April to June 2020. Field collection of these species is not prohibited in this area. The collected inflorescences were stored in plastic bottles with moistened paper.

Adult *Colocasiomyia* species that emerged from *A. odora* spadixes were collected within 24 h of eclosion. The flies were maintained in a grouped condition of approximately 20–40 flies in a plastic tube containing *Drosophila* standard yeast-based media. The male-to-female ratio in each tube was not controlled. Two *Colocasiomyia* species, *C. alocasiae* and *C. xenalocasiae*, which emerged from *A. odora*, were maintained under mixed conditions. One or 2 days before each experiment, we selected *C. alocasiae* flies under a microscope based on the bristles of the costal vein, using ice-bath anaesthesia [13,14]. We then collected *C. alocasiae* females and kept them in a group of 20 individuals.

A wild-type strain of *D. melanogaster*, Canton-S, was used for comparative experiments. Adult flies were collected within 6 h of eclosion and maintained in a grouped condition of approximately 20–40 flies. The male-to-female ratio in each tube was not controlled. One or 2 days before the experiment, we collected *D. melanogaster* females in the same manner as *C. alocasiae* females.

The mating status of the females used in the experiments was not controlled for both *C. alocasiae* and *D. melanogaster*. Flies were kept at 25°C in 40% to 60% relative humidity under a 12 h light/dark cycle. Females aged 7 to 14 days after eclosion were used for experiments.

### 2.2. Behavioural assay

Housing males and females together in a chamber increases the incidence of mating interactions. In addition, males exhibit aggressive behaviours more frequently than females, at least in *D. melanogaster* [15,16]. To avoid male–female and male–male interactions which may interfere with group formation, we used single-sex female groups for the behavioural experiments. Behavioural experiments were performed during a Zeitgeber time of 0–12 at 25°C and 40–60% relative humidity. Twenty *C. alocasiae* or *D. melanogaster* were transferred into a chamber featuring sloped walls (127 mm in diameter, 3.50 mm in depth) [17] (figure 1*b*). Video recording was performed using a CMOS camera (DFK 33UP1300, The Imaging Source Asia Co., Ltd.) equipped with a zoom lens (M0814-MP2, CBC Optics Co., Ltd.), which was initiated immediately after the fly transfer and lasted for 22 min. We used only the first 20 min of the recordings for the analyses. During the video recording, the chamber was backlit from below using a white LED array (for the white light condition) (LD-A4, HOLBEIN ART MATERIALS INC.) or infrared LED arrays (940 nm, for visual deprivation under the infrared light condition) (ISL-150 × 150-II94-BT, CCS INC.) (figure 1*b*).

Movie files recorded at 30 frames per second were analysed offline. We used Ctrax [18] to obtain trajectory data for individual flies (see Data analysis section). We performed nine replicates for *C. alocasiae* on a white LED array, 12 replicates for *D. melanogaster* on a white LED array and 11 replicates for *C. alocasiae* on an infrared LED array.

### 2.3. Data analysis

To quantify the grouping behaviour of flies, we obtained time-series data of the positions of all 20 flies in the chamber from the 20 min recorded movies by using the fly-tracking software, Ctrax [18]. In Ctrax, the silhouette of each fly was fitted to an ellipse. From the estimated position and orientation of the ellipse, the locomotion of each fly was tracked with their identity. The CSV files, which include the fly's identity, *x* position, *y* position, length of its fit ellipse, width of the ellipse, and angle of the ellipse, were exported from Ctrax. In our analysis, Ctrax successfully tracked most flies; however, in several cases, especially when the fly jumped or approached another individual at a close distance, fly identities were swapped. In this case, fly identities were manually corrected by comparing the series of images with the estimated fly identity in Ctrax. The corrected CSV files were used for subsequent analyses.

In our analysis, the position of the ellipse centre was used as the body centre or position of the fly. The distance between the body centres of two flies was used as the inter-individual distance. R software (v. 4.1.1) was used for subsequent analyses. All data and R code generated or analysed in this study are available on Dryad (http://www.datadryad.org) via https://doi.org/10.5061/dryad.bcc2fqzdq [19].

To determine whether the flies belonged to a group, we defined the proximal area of the fly of interest as the area from the centre of the body to a distance twice the body length, as described previously (electronic supplementary material, figure S1A) [10]. The mean value of body length of each species measured in this study was used as the species' body length (1.93 ± 0.12 mm for *C. alocasiae* and 2.58 ±0.20 mm for *D. melanogaster*, mean ± s.d.; *N* = 60 for each species). Flies were classified as belonging to a group when two or more other flies were within the proximal area (electronic supplementary material, figure S1A, B) at each time point during the 20 min observation period (i.e. 0≤t≤20×60 [s]); $\Delta t=30\;t_{\mathrm{frame}}=1\;[s]$). Due to small fluctuations in Ctrax estimation, the centre of the flies that did not move sometimes fluctuated, which resulted in fragmentation of the grouping duration. To avoid such artificial fragmentation, we set a threshold of 5 s: if the centre of a fly was found to have moved out of the range (the proximal area of other flies) with a duration of less than 5 s, we defined this event as ‘stay in a group’. We then defined the ‘stay event’ when a fly kept the ‘stay in a group’ condition for at least 10 s and the ‘total grouping duration’ as the total duration of stay events in each fly during the 20 min observation period.

**Figure 2.**
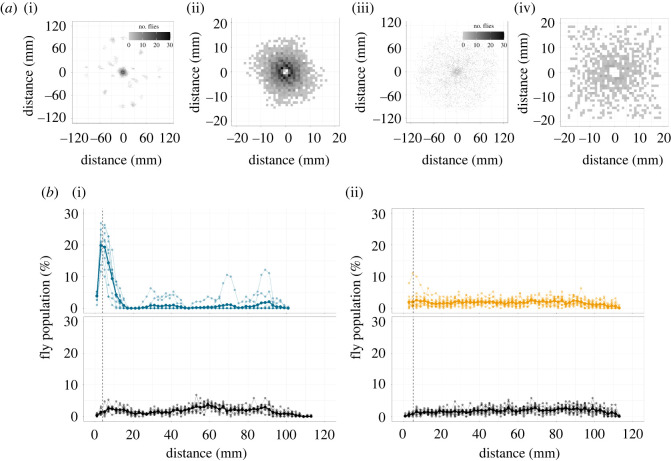
Group characteristics in an artificial chamber. (*a*) Accumulative distributions of flies at the end of the observation period (see Materials and methods). Data on *C. alocasiae* (i,ii) and *D. melanogaster* (iii, iv) are shown. In each species, the (ii,iv) show a magnified view of the (i,iii). (*b*) Two-dimensional distributions of the inter-individual distances of the fly population (top) compared to random data (bottom). Data on *C. alocasiae* (i) and *D. melanogaster* (ii) are shown. Random data for each species (bottom) were created from the distribution data of each species (top) (see Materials and methods). Thick and thin lines indicate the mean values of all chambers and those of individual chambers, respectively. Vertical dashed lines indicate the diameter of the proximal area (a distance twice the body length) to determine whether the fly is classified as belonging to a group or not.

To quantitatively compare grouping characteristics, we adopted a method described in a previous study with minor modifications [12]. In this analysis, we used the position data of all flies in a chamber (i.e. 20 flies) at the end of the observation period (i.e. 20 min after the start of the experiment, t=20×60 [s]). We first focused on a fly in a chamber and plotted the relative positions of 19 other individuals aligned with the focal individual as the origin. We repeatedly applied the same procedure to all flies in the chamber and acquired the cumulative distribution of all surrounding individuals in a single chamber. The same procedure was repeated for all experimental chambers. Here, the number of position data for the white light condition was 20 (the number of flies in a chamber) × 19 (the number of other flies in a chamber) × 9 (the number of chambers) = 3420 for *C. alocasiae* and 20 × 19 × 12 = 4560 for *D. melanogaster*. We then overlaid the plots at a bin size of 2 mm to summarize the accumulative distribution of each species or experimental condition (figure 2*a*). The maximum number of position data points in each bin obtained in this study was 30. Two-dimensional distribution maps of surrounding individuals of focal flies were generated by plotting the distribution of inter-individual distances of all the combinations of two flies in a chamber at the end of the observation period; t=20×60[s] (bin: 2 mm, number of data points: $\sum_{i=1}^{19}i=190$ combinations/chamber; figure 2*b*, top; electronic supplementary material, figure S1D, top).

To create random data, we again followed the procedure used in a previous study [12] (electronic supplementary material, figure S2A). Briefly, from the corrected CSV files of the fly tracking for each species and condition, we pooled the position data of all flies at all time points (i.e. 0≤t≤20×60 [s]); $\Delta t=30\;t_{\mathrm{frame}}=1\;[s]$) in all chambers. After alignment with the centre of the chamber, the positions of 20 individual flies were randomly selected as random data. This procedure was repeated 9 and 12 times for *C. alocasiae* and *D. melanogaster* on a white LED array, and 11 times for *C. alocasiae* on an infrared LED array, each of which corresponds to the number of replicates for each condition. These procedures produced random data for each condition (i.e. *C. alocasiae* and *D. melanogaster* on a white LED array, and *C. alocasiae* on an infrared LED array). To obtain accumulative distributions and two-dimensional distribution maps for random data, the same procedure as for the real data was applied (bin: 2 mm; figure 2*b*, bottom; electronic supplementary material, figure S1C,D, bottom).

To quantify the possibility of a fly joining a group, we defined the behavioural event of a fly encountering a group as an encountering event, which requires the following two conditions:

— The fly changes its behavioural state from ‘not belonging to the group’ to ‘belonging to the group’ (see above for definition).
— The fly walked for at least 5 s before changing state. ‘Walking’ is defined as a fly having a locomotor speed higher than a threshold (1 mm s^−1^ for *D. melanogaster*, 1 mm s^−1^ for *C. alocasiae* under the white light condition, and 2 mm s^−1^ for *C. alocasiae* under the infrared light condition, respectively). These thresholds were determined by considering the walking speed of each species and the condition (electronic supplementary material, figure S1E). The numbers of total encountering events in all conditions are presented in electronic supplementary material, tables S1 and S3.

**Figure 3.**
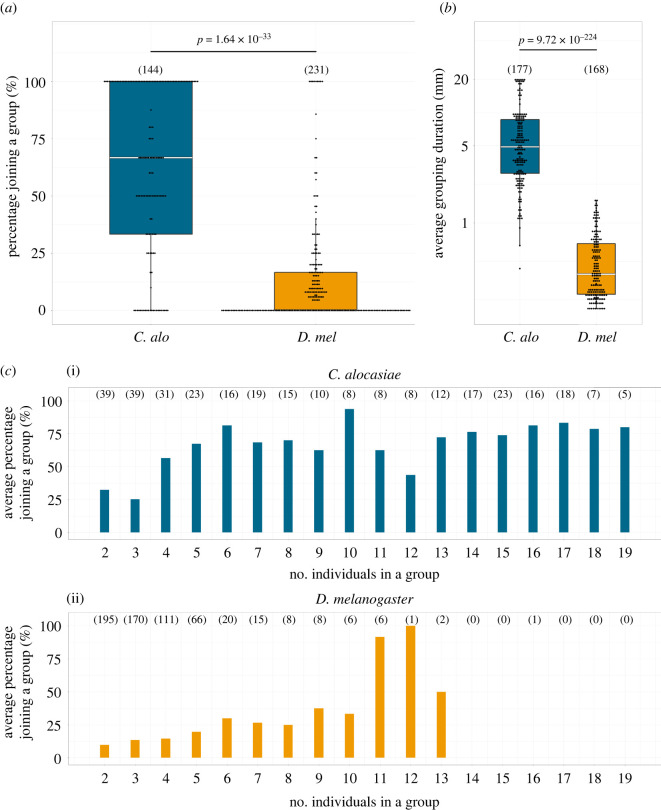
Comparison of individual behaviours during group formation. (*a,b*) Percentage of flies joining a group (*a*) and distribution of average durations stayed in a group (*b*). Each point indicates individual data. The numbers of data points are shown in parentheses. The Brunner–Munzel test was used for statistical analysis. Boxplots display the median of each group (horizontal white line in each box) with the 25th and 75th percentiles and whiskers denote 1.5× the inter-quartile range. *C. alo*, *C. alocasiae*; *D. mel*, *D. melanogaster.* (*c*) The average percentage of flies joining a group according to the group size. Data on *C. alocasiae* (i) and *D. melanogaster* (ii) are shown. The group size was defined by the number of individuals in a group which the fly encountered. The numbers of encountering events (see Materials and methods) are shown in parentheses.

For each encounter, we classified each fly's behaviour as one of two events, ‘join’ or ‘leave’. When the fly stayed in the group for 10 s or longer, we classified the event as ‘join’ (i.e. group-joining event); when the fly left the group within 10 s, we classified the event as ‘leave’. The number of group-joining events (*N*_join_) and number of total encountering events (*N*_encounter_) of each fly during the 20 min observation period were counted (electronic supplementary material, table S1). The percentage of joining a group of each fly was calculated as follows:

$$\frac{N_{\mathrm{join}}}{N_{\mathrm{encounter}}}\times 100,$$

in which only flies that had experienced encounter events were included. The percentages of joining a group of each fly were pooled and plotted for each experimental condition (i.e. *C. alocasiae* and *D. melanogaster* on a white LED array, and *C. alocasiae* on an infrared LED array) (figures 3*a* and 6*b*).

The average grouping duration of each fly that had experienced the stay events in a group was calculated by

$$\frac{\mathrm{Total}\;\mathrm{grouping}\;\mathrm{duration}\;}{N_{\mathrm{stay}}},$$

where *N*_stay_ is the number of stay events observed in this fly. The average grouping durations of all flies under each experimental condition (i.e. *C. alocasiae* and *D. melanogaster* on a white LED array, and *C. alocasiae* on an infrared LED array) were pooled and plotted (figures 3*b* and 6*c*). The total grouping duration, *N*_stay_ and mean grouping duration of each fly during the 20 min observation period are listed in the electronic supplementary material, table S2.

To quantify the sizes of the groups that the flies encountered, the positions of the grouped flies were partitioned using the x-means clustering method (https://aaaazzzz036.hatenablog.com/entry/2013/11/27/210355), and the number of individuals in the group which the fly of interest encountered was counted. The percentage of joining a group (figure 3*c*; electronic supplementary material, figure S1F) was then calculated using the dataset of the flies that had experienced the encounter events as follows. For each fly, the encountering events were first classified by the group size (i.e. the number of individuals in the group) she encountered. The group size varied between 2 and 19 [flies]. Next, the percentage that this fly joined the group at each group size was calculated. This percentage was used as a representative value of the percentage joining a group for this fly at each group size. Finally, the average values according to the group sizes were plotted for each experimental condition (figure 3*c*; electronic supplementary material, figure S1F and table S3).

**Figure 4.**
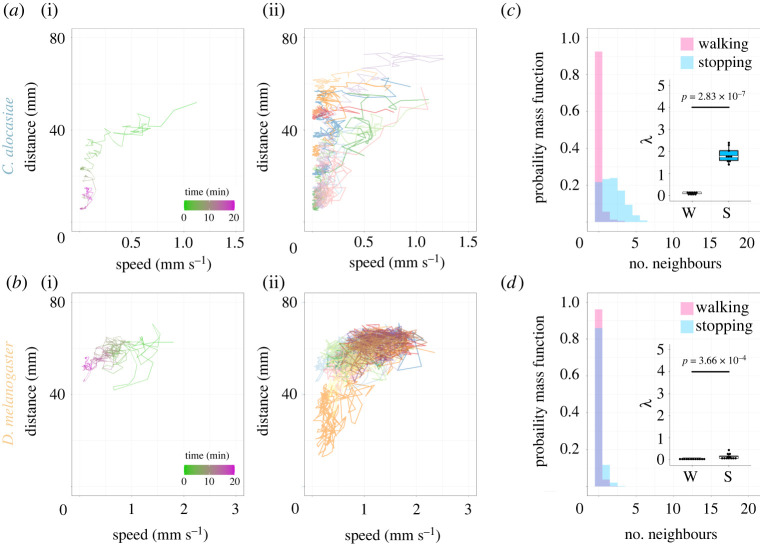
Distinct behavioural landscape between the two species. (*a,b*) Species-specific relationship between the average walking speed and average inter-individual distance. Data on *C. alocasiae* (*a*) and *D. melanogaster* (*b*) are shown; (i) indicates a representative example in each species, which is colour coded by time as indicated (light green to magenta). (ii) indicate the summary of all movie files in each species (i.e. 9 and 12 movie files for *C. alocasiae* and *D. melanogaster*, respectively), in which the speed-distance trajectory derived from each movie file is differently colour coded. The representative trajectory shown in (i) is coded in pale green (*C. alocasiae*) or pale blue (*D. melanogaster*). (*c,d*) The probability mass function in each number of the neighbouring flies in the walking (pink) and stopping (blue) states. The walking and stopping states were classified according to the locomotor speed (see Materials and methods). The overlapped areas of walking (pink) and stopping (blue) states are shown in light purple. The *λ* value shown in the inset is a parameter describing the mean of the Poisson distribution for the walking state (W) or stopping state (S). Boxplots display the medians (horizontal white line in each box) with the 25th and 75th percentiles and whiskers denote 1.5× the inter-quartile range. Each point indicates individual data. Paired *t*-test (*c*) and Brunner–Munzel test (*d*) were used. Data on *C. alocasiae* (*c*) and *D. melanogaster* (*d*) are shown.

To characterize the dynamics of fly group formation, we estimated the correlation between the average speed of flies and the average distance between flies in each chamber (figure 4*a,b*). To quantify the average speed of flies, we collected their position data at 128-frame (i.e. 4.27 s) intervals from 20 min movie files. The average speed at each time point (*t*) is described by

$$\bar{v(t)}=\frac{1}{N}\overset{N}{\underset{i=1}{\sum }}|v_{i}\;(t)|\;,$$

where $v_{i}\;(t)$ is the velocity vector of fly *i* calculated from the displacement during the observation interval $\Delta t=128\;t_{\mathrm{frame}}=4.27\;[s]$ (electronic supplementary material, figure S2B). The average distance between flies at each time point (*t*) is described by

$$\bar{d(t)}=\frac{2}{N(N-1)}\overset{N}{\underset{i,j\;(i>j)}{\sum }}d_{ij}\;(t)$$

where $d_{ij}\;(t)$ is the inter-individual distance between flies *i* and *j* at observation time *t* (electronic supplementary material, figure S2C). *N* is the number of flies in each chamber (20 flies in this study).

The correlation between the configuration and motion of flies was also estimated by taking the probability mass distributions of the number of nearest-neighbouring flies for walking and non-walking (i.e. stopping) states (figures 4*c,d* and 6*e*). We again used the position data of flies collected at 128-frame (i.e. 4.27 s) intervals from the 20 min movie files. To determine the number of nearest-neighbouring flies at each time point, we defined the surrounding area of the fly of interest as the area from the centre of the body to a distance four times the body length (electronic supplementary material, figure S2D). Notably, the results were consistent when we used five or six times the body length to define the surrounding area (electronic supplementary material, figure S3 and table S6). We then classified each fly at each time point into ‘Walking’ or ‘Stopping’ state and the probability mass distribution of each state was plotted. ‘Walking’ was defined as described previously (fly locomotor speed exceeds the threshold described above). To quantitatively compare the distributions of the number of neighbours between the walking and stopping states statistically, lambda (*λ*) values estimated from the fittings of the distribution of each chamber into the Poisson distribution by maximum-likelihood estimation were used. *λ* is the parameter describing the mean of the Poisson distribution which is given by

$$P{\;}_{\left(X=k\right)}=\;\frac{\lambda^{k}e^{-\lambda }}{k!},$$

where *k* is the number of occurrences.

### 2.4. Statistics

Statistical analyses were performed using R software (v. 4.1.1). Brunner–Munzel tests and paired *t*-tests were used for comparisons between conditions after verifying the equality of variance and normality of the values by *F*-tests and Shapiro–Wilk tests, respectively. For the Brunner–Munzel test, the brunnermunzel package (v. 1.4.1) was used (https://github.com/toshi-ara/brunnermunzel). Detailed statistical methods and results are presented in table 1; electronic supplementary material, table S6. Box plots were drawn using the R package ggplot2 (https://ggplot2.tidyverse.org/). Boxplots display the median of each group (horizontal line in each box) with the 25th and 75th percentiles, and whiskers denote 1.5 × the inter-quartile range.

**Table 1.** Summary of statistical methods and values in figures.

Figure	comparison	statistical method	statistic	statistical value	*p-*value
figure 3*a*	*C. alocasiae* - *D. melanogaster*	Brunner–Munzel test	Brunner–Munzel test statistic	−14.525	1.64 × 10^−33^
figure 3*b*	*C. alocasiae* - *D. melanogaster*	Brunner–Munzel test	Brunner–Munzel test statistic	−14.768	9.72 × 10^−224^
figure 4*c*	*C. alocasiae* [walking – stopping]	paired *t*-test	*t*	−15.606958	2.83 × 10^−7^
figure 4*d*	*D. melanogaster* [walking – stopping]	Brunner–Munzel test	Brunner–Munzel test statistic	4.93074486	0.000366
figure 6*b*	white light - infrared light	Brunner–Munzel test	Brunner–Munzel test statistic	−142.18	1.86 × 10^−36^
figure 6*c*	white light - infrared light	Brunner–Munzel test	Brunner–Munzel test statistic	−15.77	9.14 × 10^−37^
figure 6*e*	*C. alocasiae* (IR) [walking – stopping]	paired *t*-test	*t*	−4.5519305	0.001055

## 3. Results

*Colocasiomyia alocasiae* flies are known to be densely grouped in the inflorescence of their host plant, *A. odora* (figure 1*a*i*,a*ii) [20]. However, previous studies have not proven whether these flies show such aggregations under laboratory conditions. To examine this, we used a high-throughput tracking system coupled with artificial chambers developed to study the locomotion and social behaviour of *D. melanogaster* flies (figure 1*b*) [17]. For comparison, we also observed the grouping behaviour of *D. melanogaster* flies.

In our 20 min observation period, *C. alocasiae* flies grouped faster than *D. melanogaster* flies (figure 1*c*; electronic supplementary material, Movies S1 and S2). When the transition of group size (i.e. the number of flies in a group or groups) was quantified, the group of *C. alocasiae* flies grew in the first 5 min and saturated at approximately 7 min (figure 1*c*iii). Most flies remained in a group, or groups, after saturation. By contrast, the group size of *D. melanogaster* flies remained small throughout the observation period (figure 1*c*iv). These results indicate that the dynamic processes of group formation differ between these two species, and that the group size of *C. alocasiae* was larger than that of *D. melanogaster*.

To quantify the population density of a group, we plotted the cumulative distribution of all other flies for each individual. At the end of the observation period, *C. alocasiae* flies were densely clustered, whereas *D. melanogaster* flies were more scattered in loose clusters (figure 2*a*). Indeed, the two-dimensional distribution of the fly population showed a peak at approximately 4 mm from the focal individual in *C. alocasiae*, violating a random distribution pattern (figure 2*b*i). *D. melanogaster* flies, on the other hand, showed a small violation from a random distribution only near the focal individual (figure 2*b*ii). These findings indicate that *C. alocasiae* flies aggregate faster and more densely than *D. melanogaster* flies under laboratory conditions.

To identify the behavioural properties contributing to the interspecific differences in group formation, we quantified the behaviour of individuals during the process. The percentage of individuals joining the group when an individual fly encountered it was significantly higher in *C. alocasiae* than in *D. melanogaster* (figure 3*a*; table 1; electronic supplementary material, tables S1 and S4). Moreover, individuals that belonged to a group kept staying in the group longer in *C. alocasiae* than in *D. melanogaster* (figure 3*b*; table 1; electronic supplementary material, tables S2 and S5). These behavioural properties together, therefore, facilitate the formation of aggregates (i.e. dense group formation) in *C. alocasiae* flies when they encounter a group.

In the ‘optimal group size’ theory [1], the costs and benefits for the individual joining a group depend on the group size which he/she encounters, and the decision-making determined by this balance results in the difference of group size. This suggests that the group size dependency of the probability of joining a group might differ among species. To evaluate this possibility, we quantified the individual group-joining probabilities across the encountered group sizes (2–19 flies). *C. alocasiae* flies showed a high probability (greater than 50%) of joining the group when the group comprised four or more flies. By contrast, *D. melanogaster* flies demonstrated a low probability up to a group size of 10 flies and then suddenly showed an increased probability when the size reached 11 flies (figure 3*c*; electronic supplementary material, table S3). This observation indicates that the threshold for decision-making to join a group is lower in *C. alocasiae* than in *D. melanogaster*, which would affect the distinct dynamic process of group size growth.

**Figure 5.**
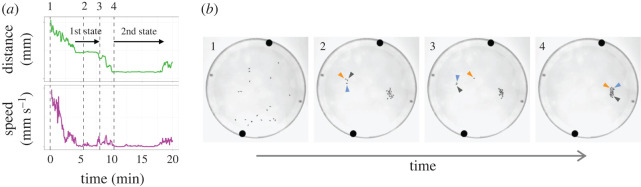
Stepwise group formation in *C. alocasiae* flies. (*a*) Time traces of the average inter-individual distance (green) and average walking speed of flies (magenta). Two stable states are marked with arrows (top). Numbers on the top indicate the time points of chamber images shown in (*b*). (*b*) The chamber images at the characteristic time points (indicated as dashed lines and numbers on top in (*a*)). Arrowheads show the flies moved from a small group to a large group. The same individuals are indicated in the same colour.

The group size dependency of group joining suggests that flies change their behaviour according to the landscape of the surrounding individuals. To quantify this property, we plotted the average walking speed of the flies against the average inter-individual distance in each chamber (figure 4*a,b*; electronic supplementary material, figure S2B–D). In *C. alocasiae*, we observed a drastic decrease in the average speed, with a relatively small decrease in the average distance (figure 4*a*). The dynamics after this initial relaxation were characterized by a rather intermittent and stepwise decrease in the average distance, during which the average speed increased slightly. This corresponds to the flies first forming initial smaller groups and the subsequent slow reorganization into a larger group (described later in detail; figure 5*a*). Such group-forming behaviour was not observed in *D. melanogaster*, which rather showed a trend of a gradual decrease in speed when the distance was decreased (figure 4*b*).

To statistically evaluate the relationships between behavioural states and the number of surrounding individuals, we classified the flies of each species into two states, walking and stopping, and compared the number of surrounding individuals (i.e. neighbours) between the two states (figure 4*c,d*). While the number of neighbours of *C. alocasiae* in the walking state (pink bars) showed a sharp peak at zero with fast decay, the number in the stopping state (blue bars) was remarkably larger and showed a broader distribution (figure 4*c*). Such a state-dependent difference in probability was not evident in *D. melanogaster* (figure 4*d*). We estimated the mean of the distributions, *λ*, by fitting the data from each movie to a Poisson distribution (see Materials and methods). In *C. alocasiae*, the *λ* values were drastically different between the two states (Cohen's d effect size = 7.25; figure 4*c* inset; table 1). In *D. melanogaster*, *λ* values were also distributed differently between the two states, although this difference was relatively small (Cohen's d effect size = 1.16; figure 4*d*, inset; table 1). These results validated our observation that *C. alocasiae* individuals located away from other individuals start walking, and stop and form a group when approaching others (electronic supplementary material, Movie S1). This tendency was weaker in *D. melanogaster* flies (electronic supplementary material, Movie S2). This unique property of *C. alocasiae* flies leads to the simplicity and stability of their group formation.

The results described above indicate that *C. alocasiae* flies initially in sparse positions move toward each other to shorten inter-individual distances and finally converge into a stable state in which most flies are located close to each other to form a dense group. However, in some datasets of *C. alocasiae* flies, we observed two stable states, instead of one, along the distance axis (figure 5*a*, top). The transition between these two stable states reflected the merging of a small group into another larger group (figure 5*b*). This explains the dynamics of the intermittent and stepwise decrease in average distance after the initial decrease in average speed (figure 4*a*). We found that the walking speed increased just before the transition and then decreased again at the end of the transition, which was manifested by a decrease in the inter-individual distance (figure 5*a*, bottom). During this process, each fly in a small group gradually started walking to leave the group and joined another group, which finally led to the collapse of the small group while fusing into a large group (figure 5*b*; electronic supplementary material, Movie S3). This observation further suggests that individual decision-making contributes to the unique group characteristics of *C. alocasiae*.

**Figure 6.**
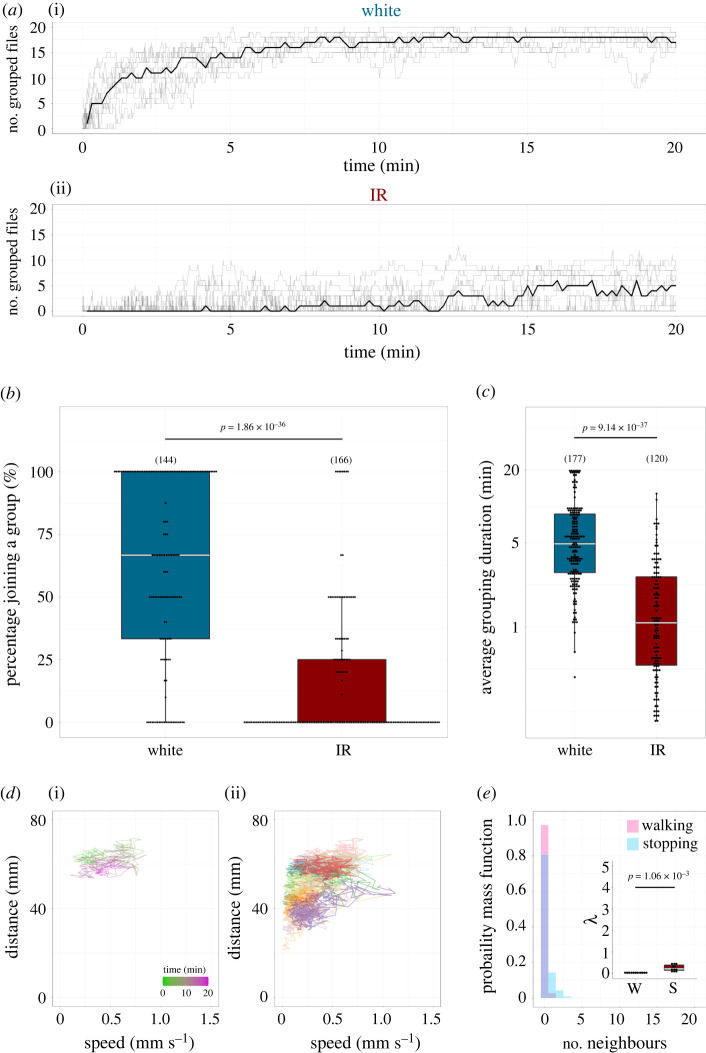
Group formation behaviour of *C. alocasiae* under the vision-deprived condition. (*a*) Time traces of the numbers of grouped flies under different light conditions. White light (i) and infrared light (IR, ii) conditions are shown. The thick line shows the median of the time traces and the thin line shows the individual traces for each chamber. (*b,c*) Percentage of joining a group (*b*) and comparison of average durations stayed in a group between the two conditions (*c*). Each point indicates individual data. The numbers of data points are shown in parentheses. The Brunner–Munzel test was used. Boxplots display the median of each group (horizontal white line in each box) with the 25th and 75th percentiles and whiskers denote 1.5× the inter-quartile range. (*d*) The relationship between the walking speed and the inter-individual distance under the IR condition. The (i) indicates a representative example, which is colour coded by time (light green to magenta). The (ii) indicates the summary of all movie files in the IR condition (i.e. 11 movie files), in which the speed-distance trajectory derived from each movie file is differently colour coded. The representative trajectory shown in the (i) is coded in pink. (*e*) The probability mass function in each number of the neighbouring flies in the walking (pink) and stopping (blue) states in the IR condition. The overlapped areas of walking (pink) and stopping (blue) states are shown in light purple. The *λ* value shown in the inset is a parameter describing the mean of the Poisson distribution for the walking state (W) or stopping state (S). Boxplots display the medians (horizontal white line in each box) with the 25th and 75th percentiles and whiskers denote 1.5× the inter-quartile range. Each point indicates individual data. The paired *t*-test was used for comparisons.

The group size dependency of group-joining probability in *C. alocasiae* suggests that they use sensory cues to detect other individuals and then decide whether to join the group. To evaluate whether visual cues were relevant in this context, we monitored the group formation of visually deprived *C. alocasiae* flies. Under an infrared (IR) light, the group formation of *C. alocasiae* flies dramatically decreased (figure 6*a*; electronic supplementary material, figure S1C and Movie S4). The group-joining probability and duration of each fly staying in a group also significantly decreased (figure 6*b,c*; table 1; electronic supplementary material, tables S1–S3 and figure S1F). The relationship between walking speed and inter-individual distance did not exhibit a typical *C. alocasiae*-like distribution under visual restriction (figure 6*d*). Under these conditions, the number of neighbouring flies showed a similar distribution between the walking and stopping states to that observed in *D. melanogaster* (Cohen's d effect size = 2.02; figure 6*e* inset; table 1). Therefore, the dense group formation and underlying decision-making properties of *C. alocasiae* flies (whether they walk or stop) largely rely on their visual capabilities.

## 4. Discussions

In this study, we established a group formation assay for *C. alocasiae* flies under laboratory conditions for the first time. *C. alocasiae* flies formed a group without any host flower cues, which facilitated the study of the dynamic process of group formation. *C. alocasiae* fly groups are characterized by fast growth and stability. The dynamic processes and individual behaviour during group formation, such as group-joining probability, grouping duration and the dependency of behavioural choice (walk or stop) on the number of neighbouring flies, were distinct from those of *D. melanogaster*. These differences resulted in significant species differences in group characteristics.

Our study found that the group size dependency of group joining of *C. alocasiae* flies was significantly different from that of *D. melanogaster*; *C. alocasiae* flies joined a group even when the group size was small (greater than three individuals), whereas *D. melanogaster* flies joined only when the size was sufficiently large (greater than 10 individuals). This finding suggests that the optimal group size, which is determined by the balance of costs and benefits of grouping, differs between these two species, and this difference affects the individual decisions that result in the species specificity of group characteristics. One possible interspecific difference in ecological traits that could explain this difference is the availability of food resources. *D. melanogaster* flies feed and breed on rotten fruits that vary in size and are shared by many other insect species under natural conditions [5,6]. *C. alocasiae* and the related *C. xenalocasiae* flies, on the other hand, are the unique and dominant feeders of *A. odora* [3,20]; the adults feed on the flower exudates and lay eggs on the flowers, which are consumed by larvae [21,22]. The dominant resource use, as well as the large size of the host plant inflorescences, suggests that food competition, a major grouping cost, is weaker in *C. alocasiae* than in *D. melanogaster*. In addition, the high population density of fly larvae caused by the dense egg-laying of adults may promote effective food consumption through cooperative behaviour [8]. Food differences might affect the optimal group size via the beneficial aspects of the grouping. Other ecological traits (e.g. predation risk, mating competition and disease frequency), which affect the costs and benefits of grouping in general, may also result in different dynamics of group formation between these species. Future studies to elucidate the natural history of *C. alocasiae,* as well as *D. melanogaster,* will provide deeper information on how each ecological trait contributes to species-specific group formation.

Animals use sensory cues to detect other individuals and decide to form groups. The group size dependency of the group joining and the obvious relationship between walking speed and inter-individual distance together indicate that *C. alocasiae* recognizes surrounding individuals to form a group by some sensory system(s). A previous study found that *D. melanogaster* uses multiple sensory modalities for group formation [12]. When visually deprived, *D. melanogaster* flies dispersed throughout the behavioural chamber and failed to form groups. In the current study, we identified a visual requirement for *C. alocasiae* group formation, indicating a shared mechanism between *C. alocasiae* and *D. melanogaster*. Differences in the size dependency to join a group between these two fly species might reflect the visual threshold to recognize the multiple flies as group-to-be-joined or not. Exploring the visual systems that underlie such threshold-based decisions is an interesting direction for future studies.

A previous study found that *D. melanogaster* flies displayed a graded decrease in freezing behaviour with increasing group size [23]. A shared mechanism to detect group size might be involved in the decision to join a group in both species. In *D. melanogaster*, the olfactory and gustatory systems also play a role in group formation. Aggregation pheromones that attract conspecifics are widely used by insects and other arthropods to mediate group formation [24,25]. *cis*-vaccenyl acetate (cVA) induces aggregation in *D. melanogaster* [26]. In gregarious desert locusts, 4-vinylanisole (4VA) has been detected as an aggregation pheromone [25]. Further studies are needed to confirm whether sensory modalities other than vision, especially aggregation pheromones, also contribute to group formation in *C. alocasiae* flies.

## Data Availability

Data are available from the Dryad Digital Repository: https://doi.org/10.5061/dryad.bcc2fqzdq [19]. The data are provided in the electronic supplementary material [27].
